# Toxicological Considerations, Toxicity Assessment, and Risk Management of Inhaled Nanoparticles

**DOI:** 10.3390/ijms17060929

**Published:** 2016-06-14

**Authors:** Shahnaz Bakand, Amanda Hayes

**Affiliations:** 1 School of Health & Society, The University of Wollongong, Wollongong NSW 2522, Australia; sbakand@uow.edu.au; 2 School of Chemistry, The University of New South Wales, Sydney NSW 2052, Australia

**Keywords:** inhalation, nanoparticles, nanomaterials, physicochemical properties, toxicity mechanism, risk assessment

## Abstract

Novel engineered nanoparticles (NPs), nanomaterial (NM) products and composites, are continually emerging worldwide. Many potential benefits are expected from their commercial applications; however, these benefits should always be balanced against risks. Potential toxic effects of NM exposure have been highlighted, but, as there is a lack of understanding about potential interactions of nanomaterials (NMs) with biological systems, these side effects are often ignored. NPs are able to translocate to the bloodstream, cross body membrane barriers effectively, and affect organs and tissues at cellular and molecular levels. NPs may pass the blood–brain barrier (BBB) and gain access to the brain. The interactions of NPs with biological milieu and resulted toxic effects are significantly associated with their small size distribution, large surface area to mass ratio (SA/MR), and surface characteristics. NMs are able to cross tissue and cell membranes, enter into cellular compartments, and cause cellular injury as well as toxicity. The extremely large SA/MR of NPs is also available to undergo reactions. An increased surface area of the identical chemical will increase surface reactivity, adsorption properties, and potential toxicity. This review explores biological pathways of NPs, their toxic potential, and underlying mechanisms responsible for such toxic effects. The necessity of toxicological risk assessment to human health should be emphasised as an integral part of NM design and manufacture.

## 1. Introduction

Nanotechnology can be described as the science of precise manipulation and design of matter at the nanoscale level of approximately 1–100 nanometers (nm). Detailed terminology and definitions related to different types of nano-objects such as nanoparticle, nanofibre, and nanoplate have been described [1,2]. Nanotechnology has grown to a multibillion dollar industry worldwide [3]. Engineered nanomaterials (NMs) with their unique physicochemical characteristics are rapidly being implemented in the various fields of medicine, pharmaceutics, biotechnology, energy production, environmental sciences, crop protection, transportation, housing, and electronics [4,5,6]. Novel engineered NMs and composites are continually emerging with the potential for significant commercial applications.

Engineered NMs can be manufactured from chemical elements such as metals, metal oxides, sulphides or selenides, carbon, polymers, and biological molecules such as lipids, carbohydrates, peptides, proteins, and nucleic acid oligomers [3]. The geometry of manufactured NMs may range from isometric NPs to one-dimensional (1D; nanofibres or nanotubes), and two-dimensional (2D; plate-like or disk-like NMs) forms (Table 1). Therefore, to represent the diversity of engineered NMs, a matrix based on the combination of geometry and chemistry can be used for the classification of engineered NMs [3].

The most commonly implemented NMs have been made of transitional metals (e.g., silver, gold), carbon (e.g., fullerenes, single and multi-walled carbon nanotubes, graphene), and metal oxides (e.g., ZnO, TiO_2_). Examples of high volume, commercial NMs include nanosilver, fullerenes, quantum dots, carbon nanotubes, and metal oxide NPs [3]. NPs have already been applied in sunscreens and cosmetic products, food additives, self-cleaning surfaces, disinfectants and clothing, batteries, fuel additives, and other products. A variety of NMs have been engineered to enhance medical devices, their functionality and reliability. Nanostructured materials may be applied to surfaces such as biomedical implants to enhance their biocompatibility, incorporated into nanostructured solids or composites to improve strength, conductivity, and durability, and fabricated into complex, active structures for chemical or biological sensors or other devices [3]. NMs have been promoted in cell and tissue engineering, development of medical devices, encapsulation and drug delivery, diagnostics, and genes [7]. An overview of applications of polymeric nano-carriers is provided for respiratory drug and gene delivery [8].

The development of NPs for biomedical applications and drug delivery is rapidly expanding. Future nanomedicine is projected to have an enormous impact on both diagnosis and treatment, primarily in the field of cancer nanotechnology [9]. The application of nanotechnology to medicine is significant due to the ability of NPs to resemble the dimensions of essential components of biological molecules, to be able to cross body membrane barriers more effectively, and to affect organs and tissues at the cellular and molecular levels. Biological responses to NMs at the protein and cellular levels differ from those observed for conventional materials. NPs and NM products may introduce enhanced biological effects at cellular and molecular target levels, but the potential pitfalls or undesired effects of NMs in biological systems are also an important issue that should not be ignored [10,11,12,13]. Although NPs such as some polymeric NPs may exhibit biocompatible behaviour in biological systems, prior to their therapeutic application, NPs should undergo a comprehensive physicochemical characterization in both dry and wet conditions. Examples of biosafety and biocompatibility investigations of specific NMs used in regenerative medicine have been reviewed [13]. These include quantum dots, silica, and polymer NPs that are mostly used for fluorescence imaging; super-paramagnetic iron oxide NPs used for magnetic resonance imaging; and gold NPs that are used for photoacoustic imaging [13].

Available research has indicated that ultrafine particles (UFPs) often induce mild yet significant pulmonary inflammatory responses as well as systemic effects [14]. Pulmonary exposure to NPs induces a greater inflammatory response compared to larger particles of conventional material at identical mass concentrations [15]. Following the exposure of human fibroblasts to ZnO and TiO_2_ NPs, cell viability was significantly reduced in a dose-dependent manner using the MTS Tetrazolium Salt assay [16]. *In vitro* exposure of A549 cells to micro- and nanosized copper(II) oxide induced cell viability reduction in a dose-dependent manner; however, NPs reduced the cellular metabolic activity more severely [17].

Serious damage to the human lung was reported in seven female workers who were exposed to polyacrylate containing NPs over a 5–13 month exposure period. All seven workers presented with shortness of breath and pleural effusions [18]. The compound that was stated to be polyacrylic ester consisted of NPs of 30 nm, confirmed by electron microscopy analysis of both the paste compound used and workplace dust. Several cases of human exposure to NPs that caused symptoms such as bronchiolitis, respiratory distress syndrome, and death have been reviewed [19]. While the potential toxic effects of NM exposure have been highlighted as a concern, the possible interactions of NMs with biological systems and the precise mechanisms of their toxic actions have yet to be understood.

As research and business both continue to focus on nanotech products, more research is also needed to assess the potential adverse outcomes of NPs. Toxicological risk assessment should be considered as an integral part of NM design and manufacture to ensure the health and safety of workers, consumers, and the environment. Nanotoxicology is a new discipline of toxicology that aims to precisely characterise the toxicity of NPs. Toxicological risk assessment of NPs involves hazard identification, dose-response assessment, exposure assessment, and risk characterisation. Both *in vivo* and *in vitro* toxicity methods are required for a comprehensive toxicity assessment of NPs. In the area of chronic toxicity testing such as anti-cancer research, *in vivo* methods are preferred. However, recent innovations in science and cell culture technology has allowed the development of alternative *in vitro* assay systems that are predictive, representative, and suitable for toxicity screening, e.g., for drug delivery of NPs. *In vitro* test methods have the potential to be implemented as screening tools to understand potential toxicity mechanisms of NPs [16,20,21].

The potential risks of NPs and NM products need to be assessed to understand the precise mechanisms responsible for such toxic effects and ultimately prevent human adverse effects. In this review, potential biological pathways of NPs and their toxic effects are identified and possible mechanisms of such effects are investigated. Further, recent advances and limitations related to the risk assessment and risk management of inhaled NPs are discussed.

## 2. Nanoparticle Exposure and Biokinetic Pathways

**Figure 1:**
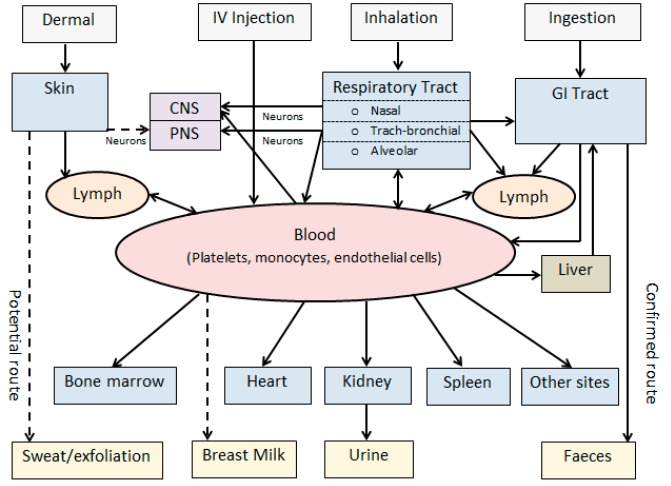
Biokinetics of nanoparticles (modified from [3]). Solid arrows: Confirmed routes; Dashed arrows: Potential routes; CNS: Central nervous system; PNS: Peripheral nervous system; GI: Gastrointestinal; IV: Intravenous.

With the exponential increase of production and commercialisation of nanotechnology-based products, the pattern of human exposure to particulates has changed significantly [4,5]. NPs may enter into the human body via inhalation, dermal, oral, and injection routes either unintentionally or deliberately (Figure 1). In either case, the ability of NPs to cross biological barriers, such as the skin, gastrointestinal (GI) tract, or blood–brain barrier (BBB), is a prerequisite for their success [9]. Research findings on dermal absorption and skin penetration of NPs are inconsistent, and more data is needed to determine whether skin provides a route of entry of NPs into the body or target tissues [22]. While injection, oral, and dermal routes may serve as important routes of deliberate entry for nanoscale therapeutic and cosmetic products, inhalation is the most significant route of entry for occupational exposures to NPs. The toxicological perspectives of inhaled therapeutics and NPs have been reviewed [21].

Inhalation and deposition of particulates in the human respiratory tract is a size-dependent cascade in which the size distribution of particles plays a major role in determining particulate airborne behaviour [20,23]. Larger particles often are impacted in the nasopharyngeal region (5–30 µm), and smaller particles (1–5 µm) are deposited in the tracheobronchial region. However, very small particles including submicron particles (<1 µm) and NPs (<100 nm) are able to penetrate deeply into the alveolar region, where the removal mechanisms are not adequate [24,25,26].

While the human respiratory tract has evolved with both structural and functional barriers to deal with inhaled particulates, it cannot always sufficiently deal with the wide range of inhaled materials [27,28,29]. Depending how deeply particles are deposited, a longer time will be required to eliminate them from the lung, and greater adverse health effects may also be expected due to particle-tissue and particle-cell interactions [29]. There is evidence that overall alveolar macrophage-mediated clearance that functions to remove inhaled particles cannot deal with NPs adequately, potentially enabling them to come into close contact with the alveolar epithelium and enter the blood stream [30,31,32,33]. NPs are too small to be efficiently recognised and phagocytised by alveolar macrophages unless they form larger particles by aggregation or agglomeration [3]. While insoluble particles may reside in the lung indefinitely [24,25,33], very small particles have the potential to cross the lung epithelial tissue barrier and readily reach other target organs through the blood stream [14].

While access to the central nervous system (CNS) is generally restricted by the BBB, inhaled NPs have the potential to cross this protective barrier and enter the brain through the blood. During inhalation, olfactory uptake is also another pathway that may provide a bypass for neuronal transport of NPs directly from the nose to the brain (Figure 1).

Potential biological pathways of NPs including routes of exposure, absorption, biodistribution, and translocation and excretory pathways are summarised in Figure 1. Following absorption and systemic translocation, NPs have the potential to bio-accumulate in peripheral organs or be excreted through faeces or urine [3,19]. The liver acts as a biological filtration system by the sequestration of 30%–99% of absorbed NPs from the bloodstream, which potentially increases toxicity at the hepatic cellular level [34]. Most NPs are known to be taken up by non-parenchymal hepatocytes. NPs that interact with hepatocytes can be cleared from the body via the hepatobiliary excretory pathway.

Once biological pathways and clearance timeframes of NPs are understood, biological monitoring methods can be developed to characterise and quantify NPs in biological systems. Such methods can be developed based on imaging techniques essential to confirm their presence and to characterise NPs in tissues associated with quantitative analytical methods [19]. In addition to biomarkers of exposure, biomarkers of effects may also be developed based on different interactions of NPs with target molecules, cells, and tissues. Toxicokinetic studies are able to identify appropriate biological samples that can be used in biometrology of NPs.

Methods applied in toxicokinetic studies of inhaled NPs have been reviewed [19]. Toxicokinetic studies suggest that NPs may preferably enter via respiratory and oral routes with possible systemic translocation, resulting in bioaccumulation in the peripheral organs or elimination via faeces and urine. An integrated two-step approach was developed to evaluate the biokinetics of inhaled NPs [35]. In this approach, a combined *in vitro* and *in silico* methods was implemented to assess the pulmonary translocation and biotransformation of gold (Au) NPs. The combination of *in vitro* and *in silico* methods was able to accurately estimate the *in vivo* biokinetics of inhaled/instilled AuNPs.

## 3. Nanotoxicity and the Potential Mechanisms

Exposure to UFPs has significantly increased respiratory and cardiovascular morbidity and mortality rates [34,36,37,38,39,40]. Combustion-derived NPs (CDNPs) are an integral constituent of urban air pollution, or PM_2.5_, and lead to these adverse health outcomes [38]. Diesel engines are a major source of particles that include high levels of NPs smaller than 50 nm [41]. CDNPs that contain both metallic and organic NPs have the potential to translocate and access the brain and other organs [42]. Ultimately, at the target sites, diverse mechanisms can possibly be responsible for their biological effects, such as the generation of reactive oxygen species (ROS), oxidative stress, mitochondrial agitation, inflammation, reticulo-endothelial uptake, protein alteration, phagocytosis impairment, endothelial malfunction, neoantigen generation, changed cell cycle regulation, and DNA damage [13,43,44].

Particle toxicity is associated with several parameters, mainly particle type, concentration and size distribution, frequency and duration of exposure, and pulmonary ventilation [25,45,46]. However, when properties of new-generation engineered NPs were compared to larger particles, significant differences were observed, such as a high number and surface area per unit volume, more reactivity, and protein and lipid absorption (corona formation) at the primary and secondary target sites [3]. The biological impacts of NPs that are related to their unique physicochemical properties can be summarised in Table 2 (modified from [20]).

Nanoscale properties that formulate NMs behaving differently are also able to affect their behaviour in biological systems [9,34]. While size distribution is the most significant parameter, other factors such as particle morphology, density, surface area, solubility, and reactivity are also essential for the evaluation of their biological interactions. Subdividing the bulk material into smaller parts can alter toxicity and explosive properties [36,37]. By decreasing the particle size, the specific surface area increases, providing a greater proportion of its atoms to be displayed on the surface [13]. Inert materials, such as TiO_2_, can become more reactive in the nanoscale range, probably due to a reduction in the size of the particles [11,14,38]. Apart from bulk chemistry, nanosize structures of a chemical may alter optical, mechanical, and electrical properties, as well as chemical reactivity, leading to different cellular uptake and interaction with biological tissues and unpredicted effects [3,9].

Nanoscale properties may change with the method of production, preparation, and storage or when introduced into the biological system [3,13]. Depending on the exposure profile and target cells, cellular responses can be minimal/reversible and can be recovered by the activation of adaptive responses, or they can be severe or irreversible and lead to a significant alteration of cellular structure and function as well as total cellular death or necrosis. The cytotoxicity of several NPs including carbon nanotubes, TiO_2_, quantum dots, and gold and silver NPs has been reviewed [12]. In addition to their physicochemical properties, the production of toxic ions, fibrous structures, high surface charge, and generation of radical species have been highlighted as potential key factors inducing cytotoxic effects.

The nanoscale size distribution of NPs plays a significant role in their potential toxicity and their ability to cross tissue and cell membrane barriers and enter into individual cells [47,48,49]. Internalized NPs may interact with different subcellular compartments [29]. For example, particles smaller than 50 nm appear to enter cells and subcellular organelles such as mitochondria and the nucleus by passive diffusion [50,51]. In addition, the very small size may lead to a direct cellular injury caused by particle-cell interactions [13]. Importantly, NPs such as CDNPs and their constituents may enter the blood or the CNS, inducing direct effects on cardiac and cerebral functions [42,52]. Metal oxide NPs (e.g., Fe_2_O_3_, Y_2_O_3_ and ZnO) have been internalized within human vascular endothelial cells in a dose-dependent manner proportional to the concentration in the culture medium [49].

Overall, clearance mechanisms of the human body are not able to deal sufficiently with nanosized materials, as there is evidence that human alveolar macrophages with the physiological function to remove inhaled particles cannot deal with NMs less than 70 nm, hence providing them with deep access into the alveolar region and the blood stream [31,33]. The phagocytic function of the alveolar macrophages was impaired following exposure to ultrafine carbon particles at 1 µg/mL and higher concentrations [30]. Although high aspect ratio NMs can be recognised by macrophages, they undergo incomplete uptake or frustrated phagocytosis [53]. Exposure of human monocytic cells to nanotubes induced phagocytosis impairment, suggesting that the ability of macrophages to remove nanofibres from the lung may be reduced [32]. CDNPs and their components have the potential to translocate to other organs [42]. At their target organs, NMs may trigger mediators and promote inflammatory or immunological responses [52]. It has been reported that SWCNTs (single-walled carbon nanotubes) translocate from the alveoli into the interstitium of the lung, promoting collagen deposition and interstitial fibrosis [54].

Nanoscale material structures have the potential to induce greater toxicity due to an expanded SA/MR available to undergo reactions. For example, the surface area of airborne 5-nm NPs is 1000 times higher than the surface area of 5-µm particles of the same chemical composition and mass concentration [3]. An increased surface area of the identical chemical may enhance surface reactivity, adsorption properties, and potential toxicity [12,55]. Animal toxicity studies have indicated that inhalation exposure to NPs can lead to more severe inflammatory responses compared to larger particles of similar composition and mass, mainly due to their surface characteristics [15]. UFPs may cause more inflammatory responses, more likely due to the large surface area [32,56,57]. An increased surface area of inhaled NPs can be sufficient to initiate inflammation [38].

The interaction of NMs with cellular chemistry can induce ROS generation and hence the generation of free radicals [10,12,15,42,58]. Free radicals are able to cause oxidative stress, tissue inflammation, and damage to cells, membranes, proteins, and DNA [31,40,42,43,44,56,57,59]. It was reported that UFPs can induce greater inflammatory responses in the rat lung compared to larger fine particles through pathways other than the release of transition metals [60]. Larger particle surface areas and increased intracellular Ca^++^ that involves oxidative stress are probably responsible for greater inflammatory responses. Oxidative stress is a common mechanism responsible for toxicity of NMs, either through direct generation of ROS at the surface of NPs or indirectly by target cells following the internalisation of NPs [3].

The cytotoxicity of silver NPs has been potentially mediated through oxidative stress, ROS generation, glutathione depletion, as well as mitochondrial membrane potential reduction [48,61]. Exposure of human pulmonary epithelial cells to SiO_2_ NPs led to ROS generation and glutathione depletion in A549 cells [62]. In addition to ROS generation, the cytotoxicity of silica NPs was found to be related to alteration of membrane integrity induced by cellular uptake [58]. The toxicity investigations of a few metal oxides in human A549 lung cells revealed that the CuO NPs were highly toxic, inducing cytotoxicity, DNA damage, oxidative injuries, and intracellular ROS [44,63]. Following the investigation of the molecular mechanisms of toxicity of different forms of Cu, poly-dispersed CuO NPs of less than 100 nm was found to be significantly more toxic than CuO NPs of 6 nm (NP6), Cu microparticles, and Cu^2+^ to A6 cells causing ROS generation, DNA damage, cell viability reduction, glutathione depletion, and eventually cell death [63].

Pro-inflammatory effects were observed in human endothelial cells following exposure to NPs of cobalt, SiO_2_, and TiO_2_, enhancing IL-8 cytokine production [47]. It has been suggested that CDNPs originating from different sources are able to mediate various adverse effects in target organs including lungs through pathways of oxidative stress, inflammation, and carcinogenesis [42]. In addition to local inflammatory effects, NPs may be translocated into blood circulation. Blood-borne particles can be delivered to secondary target organs such as the brain, heart, spleen, kidney, and liver, causing additional systemic effects [38,42].

A small size, high surface reactivity, and surface chemistry significantly contribute to the interaction of NPs with biological molecules. Toxicity analysis demonstrated that both size and surface coating of iron NPs are critical determinants of cellular response and a potential mechanism toward cytotoxicity [64]. Enhanced reactivity and distinctive surface characteristics of NMs, such as those of metallic atoms and fractal geometries, may also increase the likelihood of toxic effects [59]. High surface area and exposed surface atoms and molecules promote increased dissolution and release of ions from metallic or metal oxide NPs relative to their bulk materials. Metal ions are toxic to bacteria and aquatic organisms by the inhibition of enzymes and transport proteins [3]. For example, Ag^+^ ions released from nanosilver particles significantly accounts for the antibacterial and toxic properties of nanosilver particles [61,65]. ZnO NPs are another example of metal oxide NPs that can exhibit cytotoxicity due to the rapid release of Zn^2+^ ions.

Acute exposure of human vascular endothelial cells to NPs of yttrium or zinc oxides upregulated mRNA levels of inflammatory markers significantly; however, no inflammation was initiated by iron oxide NPs in human vascular endothelial cells [49]. Biological impacts of carbon black (CB) and TiO_2_ NPs were compared in rats, and it was concluded that ultrafine CB induced greater inflammation and epithelial damage than TiO_2_ NPs [57]. Such findings suggest that the composition and surface characteristics of NPs may significantly contribute to their biological impacts such as inflammation. Several modalities of cell death may be induced by CB and TiO_2_ NPs in human cells, inducing distinct molecular mechanisms of toxicity [66]. NPs therefore may induce cytotoxicity via several mechanisms: those that are identical may represent general pathways of nanotoxicity, and others that are specific to each NP could be associated with their specific physicochemical characteristics.

NMs have an increased potential to travel through the living organism compared to identical materials of the larger scale [42,52]. Regarding the ability of some NMs to deliver drugs throughout the human body, they may also be able to bind with and transport toxic chemicals and other pollutants. Airborne nanoparticles may work as vehicles to carry toxic chemicals and co-contaminants into the human body via the respiratory tract [24]. Significant growth in the development and production of engineered NPs can increase the potential for interactions of NMs with environmental media including the air. Carefully designed toxicity studies are required to understand the potential toxic interactions of NPs with other air toxicants such as organic compounds.

Exposure to chemical mixtures or complex atmospheres has already raised the concern of producing unexpected outcomes due to chemical or physiological interactions [26]. Multiple chemical exposures may induce a diverse range of toxic effects including respiratory symptoms and lung function defect [67]. The biological interactions of different NPs are not known, but the common pathway of oxidative stress suggests that there is a significant potential for chemical interactions such as additive or synergistic effects that need to be considered in future research [42].

Owing to their high energetic surface properties and adhesive forces, NPs may act like activated charcoal for the adsorption of other small molecules [38]. The large surface area of NPs provides a platform towards adsorption of various biological molecules including proteins, lipids, and nucleic acids. The rapid binding of proteins and NPs in biological fluids is defined as “protein corona”, but the biological consequences of protein adsorption to NPs are less clear. An example of serum protein adsorption is the binding of fibrinogen and NPs with the potential consequence of blood clot formation [68].

Biopersistency of engineered NMs, which is associated with dissolution, is an important factor influencing environmental and biological toxicity. Those NPs for which dissolution appears to be very rapid, such as ZnO NPs, biopersistence can be considered unlikely. However, in the case of extremely insoluble NPs such as carbon-based NMs, the potential for bioaccumulation inside living systems will be increased. Water solubility was found to be significantly associated with the toxicity of NPs [69]. While soluble NMs may provoke acute toxicological responses, insoluble or extremely low soluble NPs may cause a diverse range of chronic effects including carcinogenicity. Insoluble NPs have the potential to reside for years in the respiratory tract [38]. As mentioned previously, the phagocytic function of alveolar macrophages can be impaired following nanoparticle and nanofibre exposure [31,32]. Following long-term low-level repeated exposures, this property will increase the likelihood of adverse health effects. For example, CNTs that are finer than asbestos fibres, extremely strong, and highly biopersist in the lung, are likely to cause diseases (e.g., pleural mesothelioma) through similar pathogenicity [18,70,71].

NPs have a strong tendency to form aggregates and agglomerates in solid state and in many cases in liquid suspension [3]. Zeta potential is a common indicator of surface charge indicating the electrical potential at the outer surface of the spherical particle and adjacent water molecules traveling with the particle during its motion. Sodium chloride and other ions reduce zeta potential by screening the particle surface charge, often causing aggregation [3]. This property is a complicating factor in toxicity testing of NPs.

It is critical to develop an integrated strategy for toxicity testing of NMs including both *in vitro* screening and prioritisation for chronic animal testing [3]. Exploration of toxicity at the cellular and molecular levels is of great importance [61]. The concentration of NPs, particle size and size distribution, particle geometry, surface area, surface characteristics including surface coatings, the route of exposure, the duration of exposure, ion generation, protein NP corona formation, and the physicochemical characterisation of NPs are potential critical factors relating to toxicity [12,61,64,72].

## 4. Toxicity Assessment of NPs

In the US, it is expected that the comprehensive long-term toxicity testing of NPs using experimental animals would require approximately 34–53 years and the cost of $1.18 billion [73]. Therefore, it is critical to develop alternative methods in order to determine toxicology profiles of NPs. In that case, the application of *in vivo* test methods would potentially be reduced to studies such as toxicokinetics, pulmonary inflammation, and potential models of fibrosis.

A wide variety of *in vitro* assays are available to assess cellular toxicity [74]. The most frequently used *in vitro* assays to assess the cytotoxicity and biological responses of NPs have been reviewed [12,75]. Researchers often tend to implement comparatively simple *in vitro* test systems that are relatively easy to perform, control, and interpret. However, there is a need to develop validated *in vitro* assay systems for toxicity testing of an expanding range of NPs.

In the field of respiratory toxicology, *in vitro* methods have been developed using human-based cellular systems such as airway cells, lung cells or tissues, and target-specific endpoints [27,76]. Potential pulmonary toxicity of NPs can be revealed using human lung cells *in vitro* and relevant biological endpoints [74,77]. Validated *in vitro* test systems can provide readily available toxicity information relevant to inhalational NM exposure. Such methods would bring many potential benefits for the risk assessment of NPs, providing simpler, faster, and less expensive toxicity screening tools [78].

Regarding particle toxicology *in vitro* test methods using both human- and animal-based cellular systems can be employed. However, appropriate lung cell types need to be selected in order to represent significant features of its corresponding region in the respiratory tract. Physiologically relevant *in vitro* models that are able to replicate the natural cellular structure and tissue architecture of the proximal and distal parts of the respiratory tract are essential for nanotoxicology investigations [74,77].

Considering the complex nature of the architecture of the human lung, various *in vitro* models may be employed to represent the proximal and distal regions of the respiratory tract, among which cells and cell lines of the human airway (e.g., calu-3 human cell line) and alveolar epithelium (e.g., A549 human lung cells) were employed in nanotoxicology more frequently [29]. In addition, alveolar macrophages are among the first cellular systems dealing with inhaled material that can be obtained both as primary cultures by pulmonary lavage or cell lines [79]. NPs can access deeply into the distal lung where the pulmonary epithelium is composed of two distinct cell types of alveolar type 1 (AE1) and alveolar type II (AE2), serving essential functions of the alveolar epithelium. To represent the multicellular nature and complex structure of the alveolar region, promising approaches for construction of 3D alveolar tissue models *in vitro* have been reported [29,80,81,82].

*In vitro* methods can be precisely controlled; hence, they can provide more reproducible toxicity data than *in vivo* models, but require higher standardisation [29]. Considering one of the major technical challenges of *in vitro* toxicity testing of airborne contaminants, that is, to resemble inhalation exposure in cultured cells or tissues, a significant breakthrough has been achieved [28,74,77,83,84,85,86,87,88,89,90]. Human cells cultured on permeable porous membranes exposed to airborne contaminants directly at the air liquid interface (ALI) using exposure chamber systems. Direct exposure technique, which provides close contact between target cells and airborne contaminants can be applied for nanotoxicology investigations providing physiologically relevant toxicity information on NPs.

In order to allow for the efficient and homogeneous distribution of fine and ultrafine aerosols into the human cells cultured on the membrane surface, the commercially available perfusion chamber system of MINUCELL was modified [91]. The uniform particle deposition and the well-defined dose were achieved by a continuous monitoring of the particle size distribution [92].

For exposure of target cells to NPs directly at the air-liquid interface, the Karlsruhe exposure system (Vitrocell, Hannover, Germany) was implemented [93,94]. This system was equipped with a modified isokinetic sampling unit for aerosol collection from the particle-loaded air. Particles >1 µm were removed prior to exposure using a size-selective sampler such as a cyclone. A quartz crystal microbalance was also used to monitor the deposited mass per area unit to determine the accurate dose reached for target cells.

Adjustments were also made to the CULTEX (Vitrocell, Germany) system utilising inlet tubes with a hyperboloid-shaped air distribution unit constructed from Teflon or stainless steel, providing up to 80% deposition efficiency for NPs [83,95]. More recently, the CULTEX radial flow system was designed specifically for the exposure of cells to micro- and nanosized particles, implementing computational fluid dynamics (CFD) analysis to confirm an efficient, reproducible, and homogeneous deposition of particles [17]. Therefore, *in vitro* exposure methods that meet the essential requirements of the toxicity testing of airborne contaminants can be applied in parallel with real-time air monitoring techniques for toxicological risk assessments of NPs.

## 5. Risk Management of NPs

The vast diversity of engineered NMs and their great potential for commercial applications have introduced significant challenges for risk assessment and management. While the risk management framework provides a systematic and scientific approach for risk characterisation, application of such a framework to NMs often involves uncertainty factors significantly larger when compared to other chemicals or pharmaceuticals. The International Organization for Standardisation (ISO) has established a number of technical reports to provide a framework for risk assessment of nanotechnologies such as ISO/TR12855 and ISO/TR13121 [96,97]. The later technical report provides a framework for hazard identification, risk evaluation, decision options, and the risk communication of manufactured NMs in order to protect health and safety of exposed populations, including the general public, consumers and workers as well as the environment [97]. While the risk assessment framework is not unique to NMs, the process is focused on engineered NPs used in industrial settings, chemical manufacturing, and consumer product applications and on the potential risks related to the release of NMs during their life cycle. This technical report supplements issues specific to manufactured NMs and includes admitting where information is not complete, how to address information gaps, and the rationale behind risk management decisions and actions. The recommended process can be summarised in Table 3 [97,98].

Comprehensive NM characterisation is required for the risk assessment process. Therefore, different NM profiles need to be developed including physicochemical profiles, hazard profiles, and exposure profiles [97]. The unique physicochemical properties of solid materials at the nanoscale level contribute to major technical limitations. Technical limitations may induce misleading results generated by conventional toxicity assays. Several limitations have been discussed with respect to toxicity testing of NPs [3]. For example, NPs can adsorb vital dyes, cell culture micronutrients, or released cytokines due to their high surface area and hydrophobicity [75,99,100,101,102]. To prevent misinterpretation of *in vitro* data, there are significant issues that need to be addressed such as inclusion of relevant controls, assessing the ability of particles to interfere with the assays, particle dispersion, and using systematic approaches [75].

Considering toxicological and environmental studies, different types of data might be required in relation to NPs including particle characterization, potential adverse effects, detection, and quantification. While particle size distribution is a major physicochemical characteristic for toxicological studies of NPs, other important parameters include surface area, surface reactivity, water solubility, agglomeration, chemical composition, morphology as well as particle number, and mass concentrations [29,38]. Currently, high-resolution imaging techniques such as transmission electron microscopy (TEM) and scanning electron microscopy (SEM) serve as efficient tools to characterize NMs regarding their size distribution, morphology, and structure [10,44,103].

However, the efficient characterisation and quantification of NPs may require a few analytical techniques to be used together that may complicate the analysis and assessment of NMs [103]. Available methods of sampling and analytical techniques may not be capable enough to precisely quantify the concentration of NPs [38,70,104]. Collaborative and interdisciplinary research teams of nanotoxicologists, chemists, engineers, and material scientists are required to investigate and understand the NM properties and related biological interactions.

While it is possible to engineer NPs with desirable surface properties for commercial and biomedical applications, toxic effects of NMs can also be minimised using safe methods of NM design such as capping or coating of NPs [105,106]. While uncoated TiO_2_ NPs induced phototoxicity, no phototoxicity was detected in a cell assay system following coating with hydrophobic stabilisers used in sunscreen formulations [107]. As carbon nanotubes, fullerenes, and graphene are easily surface-functionalised, it has been reported that covalent functionalisation with carboxyl groups can decrease toxicity of these carbon-based NMs [105,108,109]. The therapeutic use of AgNP is diverse, ranging from dentistry, cancer treatment, and virucidal applications to biosensors in diagnosis and imaging [110]. While a dose-dependent cellular toxicity caused by AgNPs and Ag^+^ in A549 human lung-derived cells, the cytotoxicity of both silver compounds significantly decreased following pre-treatment with the antioxidant *N*-acetyl-cysteine [111]. For safe fabrication and incorporation of different shapes of AgNPs in collagen hydrogels, a method was developed recently by anchoring NPs to a thio-modified LL37 peptide [112]. Upon subcutaneous implementation, no toxic effects were observed on human endothelial and cornea epithelial cells, with no significant interleukin-6 activation. For inorganic NPs that are not cleared from the body, a possible solution is to target them to hepatocytes in order to enhance the hepatobiliary clearance [113]. To reduce NP sequestration by the liver, several strategies have been proposed such as shape, elasticity, and surface modifications of NPs. The ultimate goal of mechanistic nanotoxicology is to develop structure activity relationships that can assist the redesign of optimal and safe NMs for specific applications [3].

Exposure standards such as air quality and workplace exposure standards are recommended for airborne contaminants; however, currently very limited toxicological data makes it difficult to establish such guidelines for exposure to NPs. Considering the lack of firm toxicological and exposure profiles, the hierarchy of control measures need to be implemented for the maximum protection of human health and the environment. Strategies have been also introduced to evaluate unintentional occupational exposures to nanoparticles [114,115]. ISO has provided two technical specifications on occupational health and safety measures relevant to engineered NMs [116] and the use of the control banding (CB) approach for the occupational risk management of engineered nanomaterials [117]. CB may be applied as a qualitative strategy to assess work-related risks of NMs and to select control options [114]. CB applies categories or bands of health hazards that should be combined with exposure potentials or exposure scenarios to predict the risk level. The selection of this control approach will include a consideration of the level of risk involved. A practical guide has been recommended for industrial hygienists on managing occupational risks associated with engineered NMs, providing guidance on conducting workplace exposure assessment and risk characterization accounting methods of monitoring and describing criteria for risk management options [115].

## 6. Conclusions

While many potential benefits are expected from the development and commercialisation of NM products worldwide, the associated risks of exposure to NPs may often be ignored due to the lack of available toxicity information. Both acute and chronic adverse effects may arise following exposure to NPs and UFPs, ranging from inflammation, asthma and metal fume fever to fibrosis, chronic inflammatory lung disease, and carcinogenesis [38,42,56]. Such consequences caused by unintentional or deliberate exposures may pose health risks to exposed populations. While working populations that are engaged in the research and production of NPs may have the greatest potential for exposure, by increasing the commercial application of NM products, exposure potential for the general public is also expected to increase.

Biological interactions and toxic effects of NPs is significantly associated with their unique physiochemical properties [12,32,56]. At the nanoscale level, physical and chemical characteristics, as well as biological effects of NMs, can be changed significantly compared to the identical material of larger particles [38]. Therefore, it is likely for NPs to act like molecular-sized particles or to have hybrid properties of a molecule and a particle. Such unique nano-properties may pose a major challenge for risk assessment [59,103].

Rapidly expanding development and commercial application of NMs can increase the potential interactions of NPs with biological systems including human health. More research is required to explore the toxicity of NPs and their underlying mechanisms to support toxicological risk assessment as an integral part of nanotechnology-based product development [38,118]. Cellular uptake of NPs and cell-particle interactions are significantly determined by the physicochemical properties of NPs, experimental and exposure conditions, and cellular types [12,61,64,72]. Standardised *in vitro* methods have the potential to be applied as an initial step towards developing the toxicology profile of NPs.

There are many uncertainties regarding the risk assessment of NM exposure. However, strategies have been introduced to evaluate and manage the unintentional human exposure to nanoparticles, particularly occupational exposures. In the absence of adequate exposure and toxicological profiles, the hierarchy of control measures need to be implemented to ensure the protection of human health and to avoid underestimating the potential risks of human nanomaterial exposure.

## Figures and Tables

**Table 1:** Nano-objects; related terms and geometrical characteristics.

Nano-Objects	Nanoparticle	Nanofibre or Nanotube	Nanoplate
Geometrical Characteristics	Isometric 3 ext. dimensions in nanoscale	One-dimensional (1D) 2 ext. dimensions in nanoscale	Two-dimensional (2D) 1 ext. dimension in nanoscale
Example	Bucky ball 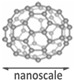	Carbon nanotubes (CNTs) 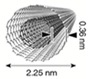	Graphene 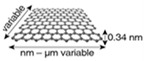

**Table 2:** Nanomaterial properties and possible biological effects.

Nanomaterial Properties	Potential Biological Effects
Size/size distribution (aerodynamic, hydrodynamic)	Crossing tissue and cell membranes
	Cellular injury
	Phagocytosis impairment, breakdown in defense mechanisms
	Migration to other organs
	Transportation of other environmental pollutants
Surface properties Surface area/mass ratio	Increased reactivity
	Increased toxicity
Chemical composition Surface characteristics	ROS generation
	Oxidative stress
	Inflammation
	Cytokine production
	Glutathione depletion
	Mitochondrial exhaustion
	Cellular injury
	Protein and DNA damage
Insolubility or low water solubility	Bioaccumulation inside living systems such as human cells, tissues and lungs
	Potential long-term effects
Agglomeration/aggregation	Interruption of cellular processes
	Cellular injury

**Table 3:** Risk management framework relevant to nanomaterials.

Risk Management	Details of Each Step Relevant to Nanomaterials
Identify hazard	Describe nanomaterial & applications
	Determine nanomaterial profiles
	Physicochemical profiles
	Hazard profiles
	Exposure profiles
Evaluate risk	Based on the combination of: Identified hazards, exposure, potential risks
	Exposure patterns: including likelihood and severity
Control risk	Level 1: Eliminate the hazard
	Eliminating the nanomaterial
	Level 2: Substitute, Isolate and engineering controls
	Substitute to a safer material, product or process
	Apply process containment
	Use local exhausted ventilation systems equipped with efficient filters (e.g., HEPA)
	Level 3: Reduce exposure by
	Administrative controls (e.g., develop Safety data sheets and safe work procedures)
	Personal protective equipment (e.g., appropriate gloves, eye and respiratory protection)
Decide, document & act	Decide: Whether or in what capacity to continue development and production of the nanomaterial
	Sharing information with the stakeholders
	Further information to be collected
Review & adapt	Update the risk assessment process through: Regular reviews
	Reviews triggered by specific events

## References

[1] ISO (2008). ISO/TR 27687. Nanotechnologies—Terminology and Definitions for Nano-Objects, Nanoparticle, Nanofibre and Nanoplate.

[2] ISO (2015). ISO/TR 80004-2. Nanotechnologies—Vocabulary—Part 2: Nano-Objects.

[3] Oberdorster G., Kane A.B., Klaper R.D., Hurt R.H., Klaassen C.D. (2013). Nanotoxicology. Casarett and Doull’s Toxicology—The Basic Science of Poisons.

[4] Renn O., Roco M. (2006). White Paper on Nanotechnology Risk Governance.

[5] Karn B., Masciangioli T., Zhang W., Colvin V., Alivisatos P. (2005). Nanotechnology and the Environment; Applications and Implications.

[6] Hougaard K.S., Campagnolo L., Chavatte-Palmer P., Tarrade A., Rousseau-Ralliard D., Valentino S., Park M.V., de Jong W.H., Wolterink G., Piersma A.H. (2015). A perspective on the developmental toxicity of inhaled nanoparticles. Reprod. Toxicol..

[7] Linkov I., Satterstrom F.K., Corey L.M. (2008). Nanotoxicology and nanomedicine: Making hard decisions. Nanomedicine.

[8] Beck-Broichsitter M., Merkel O.M., Kissel T. (2012). Controlled pulmonary drug and gene delivery using polymeric nano-carriers. J. Control. Release.

[9] Puri A. (2010). Nanoparticles: Crossing barriers and membrane interactions. Mol. Membr. Biol..

[10] Drobne D. (2007). Nanotoxicology for safe and sustainable nanotechnology. Arh. Hig. Rada Toksikol..

[11] Mazaheri M., Eslahi N., Ordikhani F., Tamjid E., Simchi A. (2015). Nanomedicine applications in orthopedic medicine: State of the art. Int. J. Nanomed..

[12] Khalili Fard J., Jafari S., Eghbal M.A. (2015). A review of molecular mechanisms involved in toxicity of nanoparticles. Adv. Pharm. Bull..

[13] Accomasso L., Gallina C., Turinetto V., Giachino C. (2016). Stem cell tracking with nanoparticles for regenerative medicine purposes: An overview. Stem Cells Int..

[14] Oberdorster G., Oberdorster E., Oberdorster J. (2005). Nanotoxicology: An emerging discipline evolving from studies of ultrafine particles. Environ. Health Perspect..

[15] Warheit D.B. (2004). Nanoparticles health impacts. Mater. Today.

[16] Dechsakulthorn F., Hayes A., Bakand S., Joeng L., Winder C. (2008). *In vitro* cytotoxicity of selected nanoparticles using human skin fibroblasts. Altern. Anim. Test. Exp..

[17] Aufderheide M., Halter B., Mohle N., Hochrainer D. (2013). The CULTEX RFS: A comprehensive technical approach for the *in vitro* exposure of airway epithelial cells to the particulate matter at the air-liquid interface. BioMed Res. Int..

[18] Song Y., Li X., Du X. (2009). Exposure to nanoparticles is related to pleural effusion, pulmonary fibrosis and granuloma. Eur. Respir. J..

[19] Rinaldo M., Andujar P., Lacourt A., Martinon L., Canal Raffin M., Dumortier P., Pairon J.C., Brochard P. (2015). Perspectives in biological monitoring of inhaled nanosized particles. Ann. Occup. Hyg..

[20] Bakand S., Hayes A., Dechsakulthorn F. (2012). Nanoparticles: A review of particle toxicology following inhalation exposure. Inhal. Toxicol..

[21] Hayes A.J., Bakand S. (2014). Toxicological perspectives of inhaled therapeutics and nanoparticles. Expert Opin. Drug Metab. Toxicol..

[22] Crosera M., Bovenzi M., Maina G., Adami G., Zanette C., Florio C., Filon Larese F. (2009). Nanoparticle dermal absorption and toxicity: A review of the literature. Int. Arch. Occup. Environ. Health.

[23] Asgharian B., Price O., Oberdorster G. (2006). A modeling study of the effect of gravity on airflow distribution and particle deposition in the lung. Inhal. Toxicol..

[24] Siegmann K., Scherrer L., Siegmann H.C. (1999). Physical and chemical properties of airborne nanoscale particles and how to measure the impact on human health. J. Mol. Struct..

[25] Rozman K.K., Klaassen C.D., Klaassen C.D. (2001). Absorption, distribution and excretion of toxicants. Casarett and Doull's Toxicology: The Basic Science of Poisons.

[26] Witschi H.P., Klaassen C.D. (2001). Toxic responses of the respiratory system. Casarett and Doull's Toxicology: The Basic Science of Poisons.

[27] Lambre C.R., Auftherheide M., Bolton R.E., Fubini B., Haagsman H.P., Hext P.M., Jorissen M., Landry Y., Morin J.P., Nemery B. (1996). *In vitro* tests for respiratory toxicity, the report and recommendations of ECVAM workshop 18. Altern. Lab. Anim..

[28] Bakand S., Winder C., Khalil C., Hayes A. (2005). Toxicity assessment of industrial chemicals and airborne contaminants: Transition from *in vivo* to *in vitro* test methods: A review. Inhal. Toxicol..

[29] Blank F., Gehr P., Rutishauser R.R. (2009). In Vitro Human Lung Cell Culture Models to Study the Toxic Potential of Nanoparticles.

[30] Lundborg M., Johard U., Lastbom L., Gerde P., Camner P. (2001). Human alveolar macrophage phagocytic function is impaired by aggregates of ultrafine carbon particles. Environ. Res..

[31] Bergeron S., Archambault D.P. (2005). Canadian Stewardship Practices for Environmental Nanotechnology.

[32] Brown D.M., Kinloch J.A., Bangert U., Windle A.H., Walter D.M., Walker G.S., Scotchford C.A., Donaldson K., Stone V. (2007). An *in vitro* study of the potential of carbon nanotubes and nanofibres to induce inflammatory mediators and frustrated phagocytosis. Carbon.

[33] Mublfeld C., Gebr P., Rutishauser B.R. (2008). Translocation and cellular entering mechanisms of nanoparticles in the respiratory tract. Swiss Med. Wkly..

[34] Kan H., London S.J., Chen G., Zhang Y., Song G., Zhao N., Jiang L., Chen B. (2008). Season, sex, age, and education as modifiers of the effects of outdoor air pollution on daily mortality in shanghai, china: The public health and air pollution in Asia (PAPA) study. Environ. Health Perspect..

[35] Bachler G., Losert S., Umehara Y., von Goetz N., Rodriguez-Lorenzo L., Petri-Fink A., Rothen-Rutishauser B., Hungerbuehler K. (2015). Translocation of gold nanoparticles across the lung epithelial tissue barrier: Combining *in vitro* and *in silico* methods to substitute *in vivo* experiments. Part. Fibre Toxicol..

[36] Dockery D.W., Pope C.A., Xu X., Spengler J.D., Ware J.H., Fay M.E., Ferris B.G., Speizer F.E. (1993). An association between air pollution and mortality in six U.S. Cities. N. Engl. J. Med..

[37] Chauhan A.J., Johnston S.L. (2003). Air pollution and infection in respiratory illness. Br. Med. Bull..

[38] Borm P.J., Robbins D., Haubold S., Kuhlbusch T., Fissan H., Donaldson K., Schins R., Stone V., Kreyling W., Lademann J. (2006). The potential risks of nanomaterials: A review carried out for ECETOC. Part. Fibre Toxicol..

[39] Ruckerl R., Schneider A., Breitner S., Cyrys J., Peters A. (2011). Health effects of particulate air pollution: A review of epidemiological evidence. Inhal. Toxicol..

[40] Stone V., Johnston H., Clift M.J. (2007). Air pollution, ultrafine and nanoparticle toxicology: Cellular and molecular interactions. IEEE Trans. Nanobiosci..

[41] Lawson G., Wang H. (2006). Public health impact of diesel exhast: Toxicity of nano-sized diesel exhaust particles—Part 1. Environ. Health.

[42] Donaldson K., Tran L., Jimenez L.A., Duffin R., Newby D.E., Mills N., MacNee W., Stone V. (2005). Combustion-derived nanoparticles: A review of their toxicology following inhalation exposure. Part. Fibre Toxicol..

[43] Nel A., Xia T., Madler L., Li N. (2006). Toxic potential of materials at the nanolevel. Science.

[44] Karlson H.L., Cronholm P., Gustafsson J., Moller L. (2008). Copper oxide nanoparticles are highly toxic: A comparison between metal oxide nanoparticles and carbon nanotubes. Chem. Res. Toxicol..

[45] David A., Wagner G.R., Stellman J.M. (1998). Respiratory system. Encyclopaedia of Occupational Health and Safety.

[46] Winder C., Winder C., Stacey N.H. (2004). Toxicology of gases, vapours and particulates. Occupational Toxicology.

[47] Peters K., Unger R.E., Kirkpatrick C.J., Gatti A.M., Monari E. (2004). Effects of nano-scaled particles on endothelial cell function *in vitro*: Studies on viability, proliferation and inflammation. J. Mater. Sci. Mater. Med..

[48] Hussain S.M., Hess K.L., Gearhart J.M., Geiss K.T., Schlager J.J. (2005). *In vitro* toxicity of nanoparticles in BRL 3A rat liver cells. Toxicol. Vitr..

[49] Gojova A., Guo B., Kota R.S., Rutledge J.C., Kennedy I.M., Barakat A.I. (2007). Induction of inflammation in vascular endothelial cells by metal oxide nanoparticles: Effect of particle composition. Environ. Health Perspect..

[50] Geiser M., Rothen-Rutishauser B., Kapp N., Schurch S., Kreyling W., Schulz H., Semmler M., Im Hof V., Heyder J., Gehr P. (2005). Ultrafine particles cross cellular membranes by nonphagocytic mechanisms in lungs and in cultured cells. Environ. Health Perspect..

[51] Chithrani D.B. (2010). Intracellular uptake, transport, and processing of gold nanostructures. Mol. Membr. Biol..

[52] Donaldson K., Stone V., Tran C.L., Kreyling W., Borm P.J. (2004). Nanotoxicology. Occup. Environ. Med..

[53] Shi X., von dem Bussche A., Hurt R.H., Kane A.B., Gao H. (2011). Cell entry of one-dimensional nanomaterials occurs by tip recognition and rotation. Nat. Nanotechnol..

[54] Wang X., Xia T., Ntim S.A., Ji Z., Lin S., Meng H., Chung C.H., George S., Zhang H., Wang M. (2011). Dispersal state of multiwalled carbon nanotubes elicits profibrogenic cellular responses that correlate with fibrogenesis biomarkers and fibrosis in the murine lung. ACS Nano.

[55] Fenoglio I., Fubini B., Ghibaudi E.M., Turci F. (2011). Multiple aspects of the interaction of biomacromolecules with inorganic surfaces. Adv. Drug Deliv. Rev..

[56] Donaldson K., Stone V., Gilmour P.S., Brown D.M., MacNee W. (2000). Ultrafine particles: Mechanisms of lung injury. Philos. Trans. R. Soc. A.

[57] Renwick L.C., Brown D., Clouter A., Donaldson K. (2004). Increased inflammation and altered macrophage chemotactic responses caused by two ultrafine particle types. Occup. Environ. Med..

[58] Kim I.Y., Joachim E., Choi H., Kim K. (2015). Toxicity of silica nanoparticles depends on size, dose, and cell type. Nanomedicine.

[59] Tolstoshev A. (2006). Nanotechnology, Assessing the Environmental Risks for Australia.

[60] Donaldson K., Brown D., Clouter A., Duffin R., MacNee W., Renwick L., Tran L., Stone V. (2002). The pulmonary toxicology of ultrafine particles. J. Aerosol Med..

[61] Dubey P., Matai I., Kumar S.U., Sachdev A., Bhushan B., Gopinath P. (2015). Perturbation of cellular mechanistic system by silver nanoparticle toxicity: Cytotoxic, genotoxic and epigenetic potentials. Adv. Colloid Interface Sci..

[62] Lin W., Huang Y.W., Zhou X.D., Ma Y. (2006). *In vitro* toxicity of silica nanoparticles in human lung cancer cells. Toxicol. Appl. Pharmacol..

[63] Thit A., Selck H., Bjerregaard H.F. (2015). Toxic mechanisms of copper oxide nanoparticles in epithelial kidney cells. Toxicol. Vitr..

[64] Sanganeria P., Sachar S., Chandra S., Bahadur D., Ray P., Khanna A. (2015). Cellular internalization and detailed toxicity analysis of protein-immobilized iron oxide nanoparticles. J. Biomed. Mater. Res. B.

[65] McShan D., Ray P.C., Yu H. (2014). Molecular toxicity mechanism of nanosilver. J. Food Drug Anal..

[66] Boland S., Hussain S., Baeza-Squiban A. (2014). Carbon black and titanium dioxide nanoparticles induce distinct molecular mechanisms of toxicity. Wiley Interdiscip. Rev. Nanomed. Nanobiotechnol..

[67] Mustajbegovic J., Zuskin E., Schachter E.N., Kern J., Vitale K., Ebling Z., Vrcic-Keglevic M. (2000). Respiratory findings in chemical workers exposed to low concentrations of organic and inorganic air pollutants. Am. J. Ind. Med..

[68] Khandoga A., Stoeger T., Khandoga A.G., Bihari P., Karg E., Ettehadieh D., Lakatos S., Fent J., Schulz H., Krombach F. (2010). Platelet adhesion and fibrinogen deposition in murine microvessels upon inhalation of nanosized carbon particles. J. Thromb. Haemost..

[69] Brunner T.J., Wick P., Manser P., Spohn P., Grass R.N., Limbach L.K., Bruinink A., Stark W.J. (2006). *In vitro* cytotoxicity of oxides nanoparticles: Comparison to asbestos, silical, and the effects of particle solubility. Environ. Sci. Technol..

[70] Seaton A. (2006). Nanotechnology and the occupational physician. Occup. Med..

[71] Poland C.A., Duffin R., Kinloch I., Maynard A., Wallace W.A., Seaton A., Stone V., Brown S., Macnee W., Donaldson K. (2008). Carbon nanotubes introduced into the abdominal cavity of mice show asbestos-like pathogenicity in a pilot study. Nat. Nanotechnol..

[72] Kettler K., Veltman K., van de Meent D., van Wezel A., Hendriks A.J. (2014). Cellular uptake of nanoparticles as determined by particle properties, experimental conditions, and cell type. Environ. Toxicol. Chem..

[73] Choi J.Y., Ramachandran G., Kandlikar M. (2009). The impact of toxicity testing costs on nanomaterial regulation. Environ. Sci. Technol..

[74] Hayes A., Bakand S., Winder C., Marx U., Sandig V. (2007). Novel *in vitro* exposure techniques for toxicity testing and biomonitoring of airborne contaminants. Drug Testing in Vitro-Breakthroughs and Trends in Cell Culture Technology.

[75] Stone V., Johnston H., Schins R.P. (2009). Development of *in vitro* systems for nanotoxicology: Methodological considerations. Crit. Rev. Toxicol..

[76] ICCVAM (2001). Report of the International Workshop on in Vitro Methods for Assessing Acute Systemic Toxicity.

[77] Bakand S., Hayes A. (2010). Troubleshooting methods for toxicity testing of airborne chemicals *in vitro*. J. Pharmacol. Toxicol. Methods.

[78] Sayes C.M., Reed K.L., Warheit D.B. (2007). Assessing toxicity of fine and nanoparticles: Comparing *in vitro* measurements to *in vivo* pulmonary toxicity profiles. Toxicol. Sci..

[79] Allen C.B., Gardner D.E. (2006). *In vitro* models for lung toxicology. Toxicology of the Lung.

[80] Mondrinos M.J., Koutzaki S., Jiwanmall E., Li M., Dechadarevian J.P., Lelkes P.I., Finck C.M. (2006). Engineering three-dimensional pulmonary tissue constructs. Tissue Eng..

[81] Lazarovici P., Li M., Perets A., Mondrinos M.J., Lecht S., Koharski C.D., Bidez P.R., Fink C.M., Lelkes P.I., Marx U., Sandig V. (2007). Intelligent biomatrices and engineered tissue constructs: *In-vitro* models for drug discovery and toxicity testing. Drug Testing in Vitro-Breakthroughs and Trends in Cell Culture Technology.

[82] Mondrinos M.J., Koutzaki S., Lelkes P.I., Finck C.M. (2007). A tissue-engineered model of fetal distal lung tissue. Am. J. Physiol. Lung Cell Mol. Physiol..

[83] Aufderheide M. (2005). Direct exposure methods for testing native atmospheres. Exp. Toxicol. Pathol..

[84] Bakand S., Winder C., Khalil C., Hayes A. (2006). A novel *in vitro* exposure technique for toxicity testing of selected volatile organic compounds. J. Environ. Monit..

[85] Bakand S., Winder C., Khalil C., Hayes A. (2006). An experimental *in vitro* model for dynamic direct exposure of human cells to airborne contaminants. Toxicol. Lett..

[86] Lestari F., Green A.R., Chattopadhyay G., Hayes A.J. (2006). An alternative method for fire smoke toxicity assessment using human lung cells. Fire Saf. J..

[87] Lestari F., Markovic B., Green A.R., Chattopadhyay G., Hayes A.J. (2006). Comparative assessment of three *in vitro* exposure methods for combustion toxicity. J. Appl. Toxicol..

[88] Bakand S., Hayes A., Winder C. (2007). An integrated *in vitro* approach for toxicity testing of airborne contaminants. J. Toxicol. Environ. Health.

[89] Bakand S., Winder C., Hayes A. (2007). Comparative *in vitro* cytotoxicity assessment of selected gaseous compounds in human alveolar epithelial cells. Toxicol. Vitr..

[90] Potera C. (2007). More human, more humane: A new approach for testing airborne pollutants. Environ. Health Perspect..

[91] Tippe A., Heinzmann U., Roth C. (2002). Deposition of fine and ultrafine aerosol particles during exposure at the air/cell interface. Aerosol Sci..

[92] Bitterle E., Karg E., Schroeppel A., Kreyling W.G., Tippe A., Ferron G.A., Schmid O., Heyder J., Maier K.L., Hofer T. (2006). Dose-controlled exposure of A549 epithelial cells at the air-liquid interface to airborne ultrafine carbonaceous particles. Chemosphere.

[93] Paur H.R., Mulhopt S., Weiss C., Diabate S. (2008). *In vitro* exposure systems and bioassays for the assessment of toxicity of nanoparticles to the human lung. J. Consum. Prot. Food Saf..

[94] Mulhopt S., Diabate S., Krebs T., Weiss C., Paur H.R. (2009). Lung toxicity determination by *in vitro* exposure at the air liquid interface with an integrated online dose measurement. J. Phys. Conf. Ser..

[95] Aufderheide M., Mohr U. (2004). A modified CULTEX® system for the direct exposure of bacteria to inhalable substances. Exp. Toxicol. Pathol..

[96] ISO (2008). ISO/TR 12885. Nanotechnologies—Health and Safety Practices in Occupational Settings Relevant to Nanotechnologies.

[97] ISO (2011). ISO/TR 13121. Nanotechnologies—Nanomaterial Risk Evaluation.

[98] Australia S.W. (2011). How to Manage Work, Health and Safety Risks: Code of Practice.

[99] Monteiro-Riveiere N.A., Inman A.O. (2006). Challenges for assessing carbon nanomaterial toxicity to the skin. Carbon.

[100] Casey A., Herzog E., Davoren M., Lyng F.M., Byrne H.J., Chambers G. (2007). Spectroscopic analysis confirms the interactions between single walled carbon nanotubes and various dyes commonly used to assess cytotoxicty. Carbon.

[101] Casey A., Herzog E., Lyng F.M., Byrne H.J., Chambers G., Davoren M. (2008). Single walled carbon nanotubes induce indirect cytotoxicity by medium depletion in A549 lung cells. Toxicol. Lett..

[102] Guo L., von Dem Bussche A., Buechner M., Yan A., Kane A.B., Hurt R.H. (2008). Adsorption of essential micronutrients by carbon nanotubes and the implications for nanotoxicity testing. Small.

[103] Englert B.C. (2007). Nanomaterials and the environment: Uses, methods and measurement. J. Environ. Monit..

[104] Cohen B.S., McCammon J. (2001). Air Sampling Instruments for Evaluation of Atmospheric Contaminants.

[105] Sayes C.M., Gobin A.M., Ausman K.D., Mendez J., West J.L., Colvin V.L. (2005). Nano-C60 cytotoxicity is due to lipid peroxidation. Biomaterials.

[106] Wysokinska E., Cichos J., Ziolo E., Bednarkiewicz A., Strzadala L., Karbowiak M., Hreniak D., Kalas W. (2016). Cytotoxic interactions of bare and coated NaGdF4:Yb^3+^:Er^3+^ nanoparticles with macrophage and fibroblast cells. Toxicol. Vitr..

[107] Schilling K., Bradford B., Castelli D., Dufour E., Nash J.F., Pape W., Schulte S., Tooley I., van den Bosch J., Schellauf F. (2010). Human safety review of “nano” titanium dioxide and zinc oxide. Photochem. Photobiol. Sci..

[108] Sayes C.M., Liang F., Hudson J.L., Mendez J., Guo W., Beach J.M., Moore V.C., Doyle C.D., West J.L., Billups W.E. (2006). Functionalization density dependence of single-walled carbon nanotubes cytotoxicity *in vitro*. Toxicol. Lett..

[109] Sasidharan A., Panchakarla L.S., Chandran P., Menon D., Nair S., Rao C.N., Koyakutty M. (2011). Differential nano-bio interactions and toxicity effects of pristine *versus* functionalized graphene. Nanoscale.

[110] Shenava A., Sharma M., Shetty V., Shenoy S. (2015). Silver nanoparticles: A boon in clinical medicine. J. Oral Res. Rev..

[111] Foldbjerg R., Dang D.A., Autrup H. (2011). Cytotoxicity and genotoxicity of silver nanoparticles in the human lung cancer cell line, A549. Arch. Toxicol..

[112] Alarcon E.I., Vulesevic B., Argawal A., Ross A., Bejjani P., Podrebarac J., Ravichandran R., Phopase J., Suuronen E.J., Griffith M. (2016). Coloured cornea replacements with anti-infective properties: Expanding the safe use of silver nanoparticles in regenerative medicine. Nanoscale.

[113] Zhang Y.N., Poon W., Tavares A.J., McGilvray I.D., Chan W.C. (2016). Nanoparticle-liver interactions: Cellular uptake and hepatobiliary elimination. J. Control. Release.

[114] Zalk D.M., Paik S.Y., Paul Swuste P. (2009). Evaluating the control banding nanotool: A qualitative risk assessment method for controlling nanoparticle exposures. J. Nanopart. Res..

[115] Ramachandran G., Ostraat M., Evans D.E., Methner M.M., O’Shaughnessy P., D’Arcy J., Geraci C.L., Stevenson E., Maynard A., Rickabaugh K. (2011). A strategy for assessing workplace exposures to nanomaterials. J. Occup. Environ. Hyg..

[116] ISO (2012). ISO/TS 12901-1. Nanotechnologies—Occupational Risk Management Applied to Engineered Nanomaterial—Part 1: Principles and Approaches.

[117] ISO (2014). ISO/TS 12901–2. Nanotechnologies—Occupational Risk Management Applied to Engineered Nanomaterial—Part 2: Use of the Control Banding Approach.

[118] Schulte P.A., Salamanca-Buentello F. (2007). Ethical and scientific issues of nanotechnology in the workplace. Environ. Health Perspect..

